# MRI-based central sarcopenia negatively impacts the therapeutic effectiveness of single-segment lumbar fusion surgery in the elderly

**DOI:** 10.1038/s41598-024-55390-1

**Published:** 2024-02-29

**Authors:** Kai Sun, Haoran Zhu, Bo Huang, Jun Li, Genjiu Liu, Genlong Jiao, Guoliang Chen

**Affiliations:** 1 Department of Orthopedic Surgery, Jiujiang University Affiliated Hospital, Jiujiang, 332006 China; 2 Department of Orthopedic Surgery, The Fifth Affiliated Hospital of Jinan University (Heyuan Shenhe People’s Hospital), Heyuan, 517400 China; 3 Department of Orthopedic Surgery, The First Affiliated Hospital of Jinan University, Guangzhou, 510630 China; 4 Dongguan Key Laboratory of Central Nervous System Injury and Repair / Department of Orthopedic Surgery, The Sixth Affiliated Hospital of Jinan University (Dongguan Eastern Central Hospital), Dongguan, 523573 China

**Keywords:** Central sarcopenia, Elderly, Lumbar fusion, Psoas and L4 vertebral index, Diseases, Medical research

## Abstract

Central sarcopenia is associated with the prognosis of various orthopedic surgeries in the elderly. This study aims to investigate its impact on the outcomes of single-segment lumbar fusion surgery in elderly patients. Retrospective analysis was conducted on 314 patients aged 60 to 80 who underwent single-segment posterior lumbar fusion surgery due to degenerative lumbar diseases. Patients were categorized into high psoas and L4 vertebral index (PLVI) and low PLVI groups according to the MRI-measured PLVI for central sarcopenia. Basic patient data, surgery-related parameters, functional assessments at preoperative and postoperative 3, 6, and 12 months, and X-ray-based fusion status were compared. The basic data of the two groups showed no significant differences. Parameters including the operative segment, preoperative hemoglobin levels, surgical duration, and intraoperative blood loss exhibited no significant variances. However, notable differences were observed in postoperative initial hemoglobin levels, transfusion requirements, and length of hospital stay between the two groups. During the postoperative follow-ups at 3, 6, and 12 months, the VAS scores for lower back pain and ODI scores in the lower PLVI group were significantly higher compared to the high PLVI group. Additionally, the EuroQoL 5D scores were notably lower in the low PLVI group. There were no significant differences between the groups in terms of leg pain VAS scores at each time point and the fusion status at 12 months postoperatively. MRI-based central sarcopenia has a negative impact on the therapeutic effectiveness following single-segment lumbar fusion surgery in elderly patients.

## Introduction

The population of individuals aged 60 and above in China increases by approximately 6.2 million annually. By defining this demographic as elderly (> 60 years), it is projected that by 2025, China will have 280 million elderly individuals, accounting for approximately one-fifth of the total population^1^. With an aging population, the prevalence of chronic lower back and leg pain due to degenerative lumbar diseases is steadily rising. Reports indicate a 62.3% increase in elective lumbar fusion surgeries in the United States between 2004 and 2015. This surge is notably higher among patients aged 65 and older (138.7%)^2^. Extensive long-term follow-up studies have demonstrated the definitive efficacy of lumbar fusion surgery for elderly individuals with degenerative lumbar changes^3^. However, ensuring surgical safety, enhancing surgical outcomes, minimizing negative impacts on the elderly, and expediting recovery remain challenging.

Previous studies have indicated the multifactorial correlation between the outcomes of lumbar fusion surgery and various factors, including patients' demographics such as age, body mass index, bone density, surgical parameters, fusion range, and fixation strength^4–6^. Recent research has highlighted sarcopenia as a potential indicator of a patient's tolerance to surgery^7–9^. Diagnostic criteria for sarcopenia have been established by scholars from Europe and Asia, encompassing assessments of muscle mass, muscle strength, and physical performance^10,11^. However, the diagnostic process for sarcopenia is arduous, involving the utilization of multiple measures and being restricted by factors such as mental state or the inability of patients to partake in testing due to pain. Therefore, there is a pressing need for a direct diagnostic method that remains unaffected by the patient’s disease status.

Recent studies suggest that diagnosing sarcopenia through Magnetic Resonance Imaging (MRI) scans of paraspinal muscles area might indicate malnutrition, restricted mobility, and overall compromised functional status^12^. However, the degree of variation in paraspinal muscle area is heavily influenced by individual variances. Surgeons are increasingly intrigued by central sarcopenia due to its objectivity and capacity to offer comprehensive insights into muscle condition. Furthermore, all patients undergoing lumbar fusion were subjected to preoperative MRI, eliminating the necessity for supplementary tests and streamlining the acquisition of pertinent information^13,14^. The psoas and L4 vertebral index (PLVI), representing central sarcopenia, has been reported to predict postoperative outcomes in elderly individuals undergoing trauma, hip or knee replacements, and pathological fractures^15–18^. However, the influence of central sarcopenia on the outcomes of lumbar fusion surgery remains unclear^19,20^. This study aims to investigate the impact of MRI-based central sarcopenia on lumbar fusion surgery outcomes in elderly patients.

## Methods

**Figure 1:**
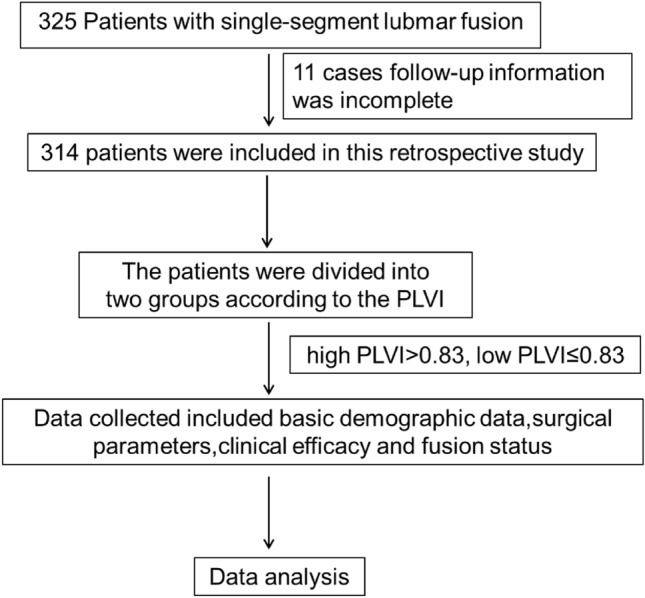
The flow chart of patient recruitment.

This study included 325 elderly patients aged between 60 and 80 years old who underwent single-segment lumbar fusion surgery from January 2017 to December 2020. Inclusion criteria were as follows: (1) diagnosis of single-segment lumbar spinal stenosis, lumbar spondylolisthesis, or lumbar spondylolisthesis combined with disc herniation; (2) surgery performed via posterior open Transforaminal Lumbar Interbody Fusion (TLIF); and (3) a follow-up period of 12 months. Exclusion criteria were: (1) history of prior spinal surgery and reoperation within one year after the current surgery; (2) scoliosis (> 10°); (3) diseases affecting bone metabolism such as chronic renal failure, hyperparathyroidism, etc.; (4) other spinal cord or spine-related diseases like spinal cord injury, epidural hematoma or abscess, or metastatic diseases (Fig. 1).

Basic demographic data (age, gender, body mass index, and bone density), surgical parameters (operative segment, preoperative hemoglobin levels, surgical duration, intraoperative blood loss, postoperative initial hemoglobin levels (24 h after surgery), transfusion requirements, and length of hospital stay), clinical efficacy (back and leg Visual Analog Scale (VAS) scores, Oswestry Disability Index (ODI) scores, EuroQoL 5D scores, and fusion status (fusion status was based on lumbar X-ray examination (CT scan if necessary), combined with modified Brantigan criteria (0–4 points), where ≥ 3 points were defined as successful fusion) at 12 months postoperatively) were collected during the follow-up period.

### Radiological assessment

**Figure 2:**
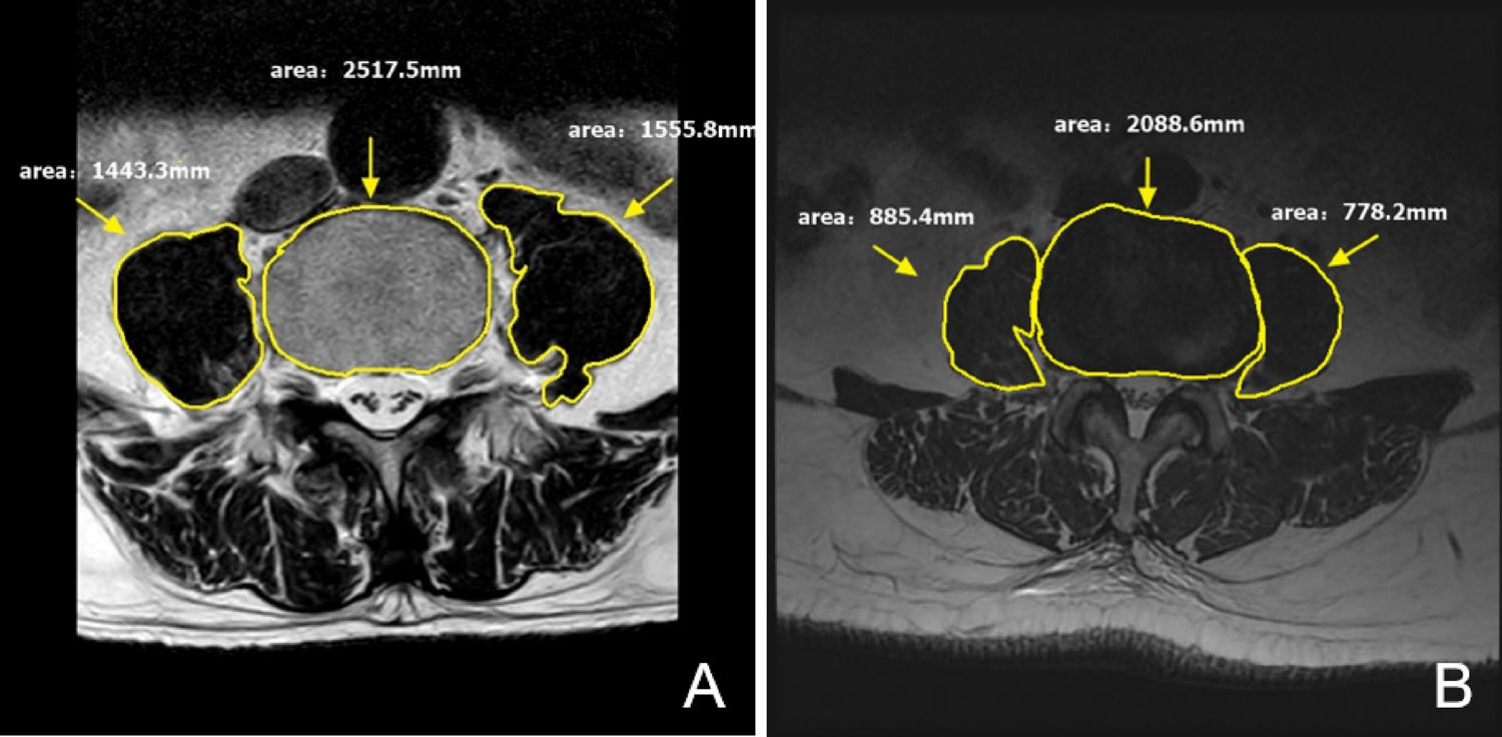
The measurement and calculation of PLVI. (**A**) the representative case of High PLVI, (**B**) the representative case of Low PLVI. PLVI = [right PCSA + left PCSA]/ L4 vertebral CSA. PLVI indicates psoas and L4 vertebral index, CSA indicates cross-sectional area, PCSA indicates psoas CSA.

**Figure 3:**
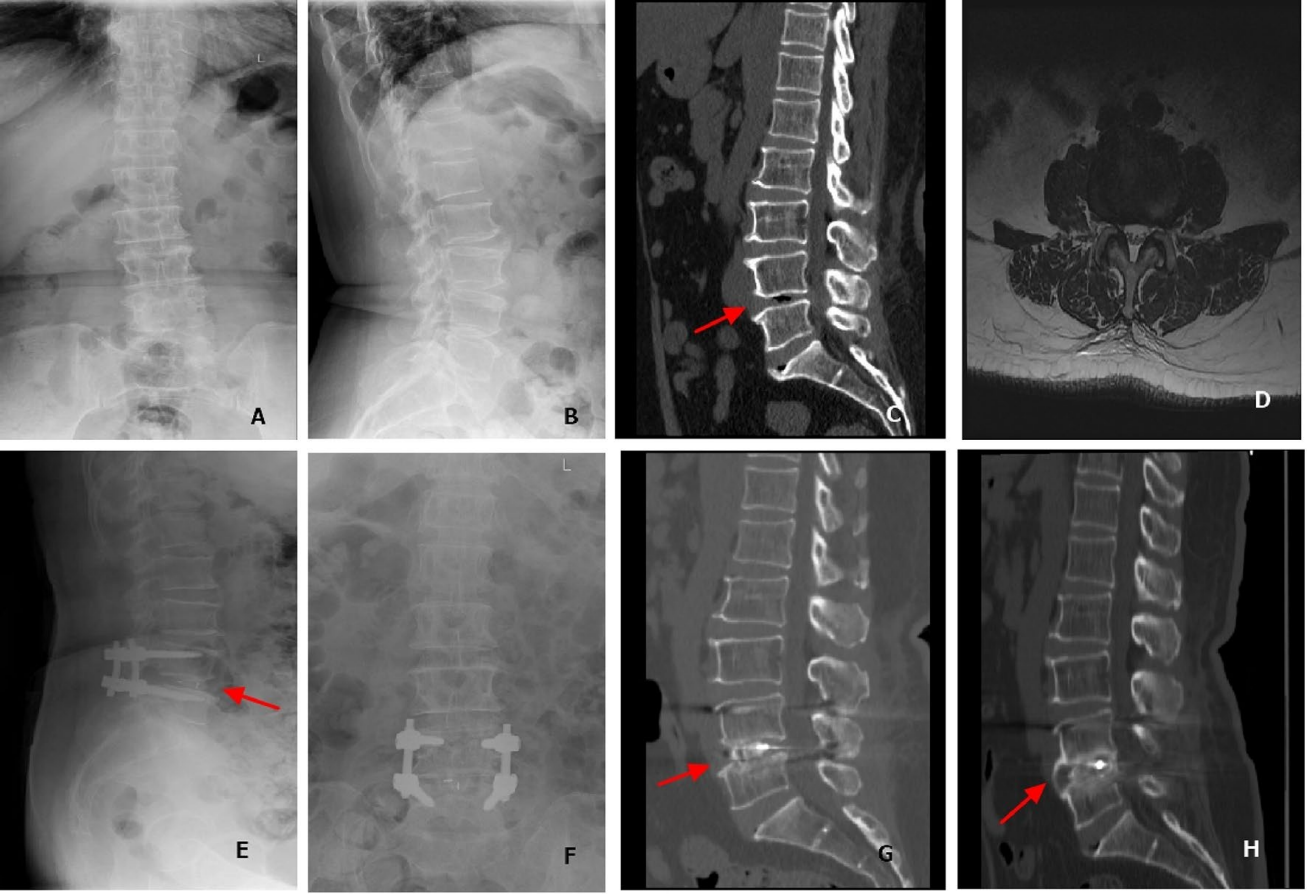
Typical case of TLIF, a 64-year-old female patient. On April 15, 2019, leg and back pain for 3 years, and DR (**A**, **B**), CT (**C**) and MR (**D**) were performed. After admission, L4-5 TLIF was performed, and DR was reexamined after surgery (**E**, **F**). 3 months later, examination of CT (**G**), 12 months later, examination of CT (**H**). Bone bridge growth ahead arrow sign.

Using the Picture Archiving and Communication System (PACS) within the hospital, positioning was performed for T2-weighted MRI at the lower edge of the L4 vertebral endplate level to measure the total cross-sectional area (TPA) of the bilateral psoas and the vertebral body area at that level. All measurements were conducted by three radiologists with specialized training. Image J software (U.S. National Institutes of Health, Bethesda, MD, USA) was utilized to process MRI to obtain the psoas and L4 vertebral index (PLVI)^18^. The PLVI, representing the ratio of the average cross-sectional area of the lumbar muscles at the lower edge of the L4 vertebral endplate level to the vertebral body, was determined. The PLVI value at the 50th percentile was established, and patients were categorized into two groups based on their relationship to the median value in the cohort: high (> 0.83) and low (≤ 0.83). (Figs. 2, 3).

### Surgical procedure

After successful endotracheal intubation and induction of general anesthesia, the patient was placed in a prone position with the abdomen suspended. Using a C-arm X-ray machine for localization of the lesion gap, a posterior midline approach was adopted, with an incision length of approximately 10 cm. Layer by layer dissection exposed the spinous process, vertebral plate, and lateral aspect of the facet joint. Under the guidance of the C-arm X-ray machine, bilateral pedicle screws (4 in total) were implanted. Segmental laminectomy, resection of the ligamentum flavum, and facet joint at the upper and lower levels were performed. Gentle retraction of the dural sac and nerve roots was followed by annulus fibrosus incision, thorough removal of intervertebral disc tissue, and cartilage scraping to induce bleeding in the endplate. Autologous bone grafting was used to fill the anterior part of the intervertebral space, compacted, and supplemented with a suitably sized cage. The pedicle screws were interconnected with rods and appropriately compressed. Negative pressure drainage was placed, followed by layered closure. The procedure was performed by an experienced senior consultant whose cumulative experience in lumbar fusion surgeries exceeded 1000 cases.

### Statistical analysis

The data processing and analysis were performed using SPSS 25.0 statistical software. The independent variables encompassed age, gender, body mass index, and bone density. Surgery-related parameters (operative segment, surgical duration, intraoperative blood loss, preoperative hemoglobin levels, postoperative initial hemoglobin levels, transfusion requirements, and length of hospital stay) and clinical outcomes (back and leg Visual Analog Scale (VAS) scores, Oswestry Disability Index (ODI) scores, EuroQoL 5D scores, and fusion status at 12 months postoperatively) were included. For univariate analysis, comparison between groups for categorical variables was conducted using Fisher's exact test, while numerical variables were assessed using the Wilcoxon signed-rank test. A significance level of P < 0.05 was considered statistically significant.

### Ethical approval and informed consent

This research protocol was approved by the Institutional Review Board of Jiujiang University Affiliated Hospital and The First Affiliated Hospital of Jinan University. The clinical procedures adhered to the principles of the declaration of Helsinki. Informed consent was obtained from all individual participants included in the study.

## Results

Out of the reviewed 325 patients, a total of 314 patients met our criteria and were included in the study for retrospective analysis. Among them, the high PLVI group comprised 169 patients, while the low PLVI group included 145 patients. There were no significant differences (P > 0.05) observed between the two groups in terms of age, gender, body mass index (BMI), bone mineral density (BMD), preoperative hemoglobin levels, surgical duration, operative segment, and intraoperative blood loss. However, notable differences were observed in postoperative initial hemoglobin levels, transfusion requirements, and length of hospital stay, demonstrating statistical significance (P < 0.05) (Table 1).

**Table 1:** Comparisons of baseline data and Perioperative parameter.

	High PLVI n = 169	Low PLVI n = 145	P value
Baseline data			
Age at surgery (year)	69.6 ± 8.2	71.8 ± 10.8	0.90
Gender (male/female)	75/94	66/79	0.94
BMI	22.7 ± 5.8	20.2 ± 4.2	0.65
BMD	− 2.2 ± 0.6	− 2.4 ± 0.3	0.76
Perioperative parameter			
Operation time (min)	104.6 ± 10.6	96.3 ± 12.9	0.63
Blood loss (ml)	110.2 ± 22.3	98.8 ± 15.7	0.54
Preoperative Hemoglobin (g/L)	132.8 ± 16.2	135.4 ± 14.9	0.86
Postoperative Hemoglobin (g/L)	96.3 ± 12.5	75.3 ± 14.5	0.02
Blood transfusion volume (ml)	54.6 ± 14.6	159.2 ± 21.3	0.0001
Blood transfusion rate (%)	29.5	48.9	0.02
Hospital stays (d)	9.8 ± 4.9	15.5 ± 5.4	0.01
Number of surgical segments			
L2-3	5	10	0.43
L3-4	22	17	0.76
L4-5	78	69	0.78
L5-S1	64	49	0.59

Comparisons of preoperative back pain VAS (6.3 ± 0.4 vs 6.7 ± 0.2), leg pain VAS (7.5 ± 1.9 vs 7.7 ± 1.5), and ODI (66.8 ± 12.5 vs 67.5 ± 15.3) scores between the two groups revealed no statistically significant differences (P > 0.05). However, during the three postoperative follow-ups, the high PLVI group exhibited better back pain VAS scores and ODI scores compared to the low PLVI group, while the EuroQoL 5D scores were relatively lower in the low PLVI group (P < 0.05). Nevertheless, there were no significant differences between the two groups in terms of leg pain VAS scores (P > 0.05) (Table 2). At the 12-month follow-up, three cases in the high PLVI group and two cases in the low PLVI group showed non-fusion, with no statistically significant difference observed (P > 0.05).

**Table 2:** Comparisons of surgical outcomes.

Group	Preoperative	Postoperative 3 months	Postoperative 6 months	Postoperative 12 months
Back pain VAS score				
High PLVI (n = 169)	6.3 ± 0.4	3.2 ± 0.8	1.3 ± 1.2	0.5 ± 1.4
Low PLVI (n = 145)	6.7 ± 0.2	4.9 ± 1.2	2.5 ± 1.5	1.9 ± 0.7
P value	0.91	0.02	0.006	0.003
Leg pain VAS score				
High PLVI (n = 169)	7.5 ± 1.9	2.5 ± 1.2	1.8 ± 1.3	0.5 ± 1.4
Low PLVI (n = 145)	7.7 ± 1.5	2.9 ± 1.6	2.2 ± 1.7	0.7 ± 1.2
P value	0.94	0.81	0.85	0.89
ODI score				
High PLVI (n = 169)	66.8 ± 12.5	32.6 ± 10.9	20.7 ± 12.4	18.4 ± 13.5
Low PLVI (n = 145)	67.5 ± 15.3	42.7 ± 11.7	28.6 ± 13.2	24.5 ± 10.8
P value	0.95	0.01	0.04	0.02
EQ-5D score				
High PLVI (n = 169)	0.52 ± 0.5	0.74 ± 0.2	0.82 ± 0.5	0.89 ± 0.7
Low PLVI (n = 145)	0.51 ± 0.3	0.62 ± 0.7	0.71 ± 0.4	0.81 ± 0.3
P value	0.93	0.04	0.03	0.04

## Discussion

Elderly patients demonstrate a notable prevalence of sarcopenia, reported to range between 14.1% and 55.9% in specific research studies^21,22^. Research indicates that variations in Asian ethnic traits, body compositions, and dietary patterns contribute significantly to an increased vulnerability to muscle loss^23^. Research on sarcopenia carries significant weight in public health, geriatrics, and gerontology. However, the diverse definitions and diagnostic parameters currently in use hinder a comprehensive understanding of the severity of sarcopenia among the elderly^22^.

In this retrospective study, the assessment of central sarcopenia using PVLI as a reference aimed to gauge its influence on elderly patients undergoing single-segment lumbar fusion surgery. The results revealed that sarcopenic patients encountered extended hospitalization, higher postoperative transfusion requirements, and inferior postoperative functional recuperation in contrast to their non-sarcopenic counterparts. Previous research has identified various factors, including advanced age, overall health status, postoperative complications, fusion level, and postoperative hemoglobin levels, contributing to prolonged hospital stays following spinal surgery^24,25^. Recent research increasingly correlates sarcopenia with extended hospitalization duration^26,27^. A study assessing sarcopenia via grip strength measurement revealed its impact on the duration of hospitalization for patients undergoing lumbar fusion surgery. Non-sarcopenic patients averaged a hospital stay of 9.4 days, while sarcopenic patients prolonged their stay to 13.4 days. This study advocates for routine grip strength assessments prior to lumbar fusion surgery and proposes preoperative interventions for sarcopenic patients to mitigate hospitalization duration and associated costs^28^. There are constraints in quantifying muscle strength in pathological circumstances. More experts are of the opinion that the utilization of imaging techniques to evaluate muscle area-related parameters holds the potential to offer a more objective evaluation of sarcopenia. A study underscored a substantially heightened risk of readmission within six months among sarcopenic patients, leading to tripled rates of hospitalization and overall costs when compared to their non-sarcopenic counterparts^29^. Our retrospective study revealed a noteworthy escalation in hospitalization duration within the low PLVI group, with no significant variances observed in preoperative age, gender, or physical status between the two groups. This underscores the impact of sarcopenia, represented by PLVI, on the hospitalization duration of lumbar fusion patients. Research indicates a spectrum of factors, both positive and negatively regulating muscle health. When the influence of positive regulators weakens, such as nutrition and exercise, and the impact of negative regulators intensifies, like inflammatory responses and trauma, adverse effects on muscle tissues become apparent^30^. The ability of post-lumbar spine surgery patients to synthesize proteins decreases, leading to increased postoperative bodily consumption and causing slow recovery and delayed discharge^31^. Another study demonstrated that the inflammatory response following surgical trauma diminishes the intake of food, thereby causing malnutrition and heightened muscle depletion in the elderly population. Consequently, this culminates in extended hospital stays^32^.

Studies indicate a heightened postoperative transfusion rate among thoracolumbar surgery patients with sarcopenia, presenting a 2.1 times greater likelihood of requiring transfusion compared to non-sarcopenic patients^33^. Similarly, another study established a link between preoperative assessment of lumbar muscle area for sarcopenia and subsequent intensive care duration and post-surgery transfusion rate^34^. In our study, both groups exhibited no significant irregularities in preoperative hemoglobin levels or intraoperative bleeding volume. However, the low PLVI group displayed a notable postoperative decline in hemoglobin levels and a marked increase in transfusion requirements. The authors propose that sarcopenic patients may undergo more concealed blood loss during and after surgery, despite limited reports addressing its origins. This phenomenon could be attributed to skeletal muscles possessing relatively higher vascularity and density compared to fat tissues. With decreased capillary density in sarcopenic patients, the total blood volume decreases, leading to a heightened relative blood loss. This perspective finds support in a study linking sarcopenia with post-head and neck tumor resection transfusion rates^35^. Hence, vigilant monitoring of postoperative hemoglobin fluctuations in elderly sarcopenic patients undergoing lumbar fusion surgery is advisable to enable early intervention and mitigate potential consequences stemming from diminished blood volume.

Descriptions of outcomes following lumbar fusion surgery in sarcopenic patients exhibit variation, yet generally acknowledge the procedure's efficacy among the elderly^36–38^. Some studies propose that sarcopenia does not substantially impact the clinical success rates of degenerative lumbar spondylolisthesis fusion surgery^39^. Correspondingly, research conducted by Japanese scholars highlighted a high prevalence of sarcopenia among Japan's elderly population, correlating it with adverse clinical outcomes post-lumbar spine surgery^40^. Our study findings indicated superior clinical outcomes in the high PLVI group compared to the low PLVI group. This disparity may be ascribed to two key factors: the diminished stability of the fusion segment's anterior column and escalated stress on adjacent segments. Muscles previously ruptured contribute to lumbar stability; superficial fibers govern spine direction while deep fibers control intervertebral shearing and torsion^41^. Studies indicate that back pain is often associated with fat infiltration in previously ruptured muscles, and increased fat infiltration in these muscles may exacerbate lumbar disc degeneration^42^. Posterior open surgery involves detaching posterior ruptured muscles, resulting in significantly reduced muscle area postoperatively and a heightened degree of fat infiltration^43^. When anterior muscles atrophy, reducing muscle area, the relative lack of muscle stability in the anterior column may induce minor postoperative lumbar instability causing back pain. Comparing pre-and postoperative anterior bone bridges at the fusion segment in our patient cohort, the low PLVI group exhibited a higher incidence of bone bridge formations than the high PLVI group, indirectly suggesting compromised anterior column stability in sarcopenic patients. A study assessing paraspinal fat degeneration using preoperative magnetic resonance imaging for sarcopenia affirmed its influence on the development of proximal junctional kyphosis and proximal junctional failure following thoracolumbar fusion surgery^44^. Another study measuring PLVI found sarcopenia to be an independent risk factor for adjacent segment degeneration in patients undergoing lumbar fusion surgery for degenerative diseases^45^. A study on adjacent intervertebral disc motion after lumbar fusion surgery suggested increased motion in adjacent segments^46^. Consequently, increased postoperative adjacent segment degeneration might result in less-than-ideal symptom relief.

### Limitation

Previous studies have discussed the effectiveness of lumbar fusion surgery, yet numerous factors affecting its outcome lack a unified conclusion. This study suffers from limitations such as a small sample size, a relatively short duration and no physical measurement of muscle strength, necessitating more detailed and precise research to validate this hypothesis.

## Conclusion

Central sarcopenia referenced by PLVI negatively impacts the surgical outcomes of single-segment lumbar fusion surgery. Before performing lumbar fusion surgery on elderly patients, evaluating sarcopenia using MRI-measured PLVI, which is simple and easily accessible, can identify sarcopenia quickly, and help to screen patients with adverse surgical outcomes.

## Data Availability

The datasets used and/or analysed during the current study available from the corresponding author on reasonable request.
